# Real-world comparison of mechanical thrombectomy vs. catheter-directed thrombolysis for the treatment of pulmonary embolism: a single-center retrospective study

**DOI:** 10.3389/fcvm.2025.1711473

**Published:** 2026-01-13

**Authors:** Isaac Demaree, Travis Pebror, Adam Schmitz, Reid Masterson, Sabah Butty

**Affiliations:** Interventional Radiology, Department of Radiology and Imaging Sciences, Indiana University School of Medicine, Indianapolis, IN, United States

**Keywords:** catheter—directed thrombolysis, mechanical thrombectomy, pulmonary artery thrombectomy, pulmonary embolism, VTE (venous thromboembolism)

## Abstract

A recent multi-site randomized controlled trial, PEERLESS, demonstrated superiority of FlowTriever mechanical thrombectomy (FTMT) to catheter-directed thrombolysis (CDT) in the treatment of intermediate-risk pulmonary embolism using a 5-point composite outcome. The purpose of our study is to review clinical outcomes between these two procedures in actual clinical practice without the use of a composite endpoint using a large sample size. This is a retrospective, single-center analysis of 461 patients who presented with submassive or massive PE and underwent treatment with either FTMT or CDT. No significant difference was observed in 7-day (RR 0.495; *p* = 0.25) or 30-day mortality (RR 1.347; *p* = 0.67). There was significantly less risk of procedure-related decompensations within the FTMT group (RR 0.221; *p* = 0.01). Non-procedure-related decompensations were similar between treatment options (RR 1.091; *p* = 0.51). ICU LOS was significantly lower for FTMT (mean 1.87 vs. 3.07; *p* < 0.001) however total LOS was longer within the FTMT (mean 6.85 vs. 5.68; *p* = 0.008). Similar to PEERLESS, we observed decreased ICU utilization with FTMT in our real-world retrospective data, likely reflecting our practice of ICU admission during thrombolytic administration. We observed slightly lower risk of procedure-related decompensations within the FTMT group, suggesting lower periprocedural morbidity; however, overall mortality between groups was not different.

## Introduction

Venous thromboembolism, including deep vein thrombosis and pulmonary embolism (PE), is the third leading cardiovascular cause of death (1). Increased pulmonary artery pressure and right heart strain reflect the severity of hemodynamic impairment by embolus and increase the risk of death (2). PE mortality is significant and can be as high as ∼52% in patients presenting with hemodynamic instability. Treatment for PE can include systemic therapies such as anticoagulation or thrombolysis, targeted therapies including catheter-directed thrombolysis and mechanical thrombectomy, and surgical options like embolectomy.

PE can be stratified based on the severity of the presentation according to the American Heart Association (AHA) and the European Society of Cardiology (ESC) guidelines (3). These guidelines delineate low-risk, intermediate-risk (ESC)/submassive (AHA), and high-risk (ESC)/massive (AHA) PEs. The stratification is determined by hemodynamic stability in addition to evidence of right heart strain or cardiac injury. High-risk/massive PEs have hemodynamic instability evidenced by either low systolic blood pressure, requirement of vasopressor support, or end-organ evidence of shock. Sub-massive/intermediate-risk PEs present with evidence of right heart strain and/or cardiac injury without shock physiology. Low-risk PEs do not meet either of the above criteria and are associated with lower mortality rates (4).

Systemic anticoagulation is the mainstay of PE treatment, which typically involves parenteral anticoagulation during the acute presentation and transition to oral agents for an extended period thereafter (5). Outcomes in patients presenting with massive PE treated with anticoagulation are relatively poor due to obstructive shock and prolonged right ventricular strain. This led to the search for a rapid method of relieving right heart strain in patients presenting with signs of hemodynamic instability. The MAPPETT-3 trial showed superiority of heparin plus thrombolytics compared to heparin plus placebo in both hospital deaths and 30-day event-free survival in patients presenting with intermediate risk PE (6). Subsequently, the PEITHO trial suggested increased risk of major hemorrhage and stroke in patients given fibrinolysis, although overall mortality was decreased (7). Catheter-directed thrombolysis (CDT) was developed as a potential method to deliver high doses of thrombolytics locally within the pulmonary artery in hopes of resolving the clot without increased risk of intracranial hemorrhage. The ULTIMA trial showed that CDT with anticoagulation decreased the RV/LV ratio more than anticoagulation alone without increased risk of bleeding (8). SEATTLE II also showed that CDT reduced RV/LV ratio with no incidents of ICH and only one major bleeding event in 150 cases (9). The OPTALYSE trial again showed low risk of major bleeding (4%) and that lower doses of thrombolytics for shorter durations effectively lowered RV/LV (10). This method has limitations such as institutional requirements to stay in the ICU while receiving therapy and the remaining concern for adverse bleeding events.

The most recent development in the treatment of acute PE is the advent of mechanical thrombectomy (MT) devices to physically remove the clot and entirely avoid the increased risk of bleeding attributable to thrombolytics. Indeed, the FLARE trial showed that mechanical thrombectomy was also effective at lowering RV/LV ratio and only 1 out of 106 patients experienced a major bleeding event (11). EXTRACT-PE, a single-arm, prospective study, also showed effective lowering of RV/LV and low (1.7%) risk of major bleeding events with thrombectomy (12). An analysis of the FLASH registry suggested low rates of adverse events (1.8%) with favorable changes in hemodynamic measures and low mortality (13). Despite the theoretical risk of decreased bleeding of MT, observational studies comparing MT against CDT have not demonstrated significant differences in mortality or bleeding risk in patients with intermediate- and high-risk PE (14, 15).

The PEERLESS trial is the first randomized controlled trial (RCT) to compare MT vs. CDT (16). Patients presenting with intermediate risk PE were randomized to either therapy. The study demonstrated superiority of the Inari FlowTriever mechanical thrombectomy (FTMT) device to CDT in a 5-point composite endpoint comprised of all-cause mortality, intracranial hemorrhage, major bleeding, clinical deterioration and/or escalation to bailout and post procedural intensive care unit (ICU) admission, however there was no significant difference between the two therapies when ICU admissions were removed from the composite endpoint. Fewer patients undergoing FTMT experienced clinical deterioration and/or bailout compared to CDT (5.4% vs. 1.8%, *p* = 0.04). There were no significant differences between the two therapies with regard to other secondary endpoints, including all-cause mortality, intracranial hemorrhage, or major bleeding (16). The findings suggest that FTMT provides a more rapid and perhaps reliable means of treating PE when compared to CDT; however, the theoretical benefit of lower risk of hemorrhage simply remains theoretical when considering these results in light of previous low-powered retrospective studies.

## Methods

### Study design and population

Periprocedural and clinical data was retrospectively reviewed on all adult patients who were treated with either FTMT or CDT at a single, large academic health center by one of several interventional radiologists. The decision of whether to intervene or not was determined by a multidisciplinary Pulmonary Embolism Response Team (PERT). Once the decision to intervene was made, the choice of FTMT or CDT was determined by availability of either device or preference of the operator. CDT was largely supplanted by FTMT within our institution by late 2019. Data from consecutive cases of CDT were included from December 2012 to June 2019 and consecutive cases of FTMT from September 2019 to June 2023.

PE diagnosis was confirmed with computed tomography angiography (CT) in most cases. Ventilation/perfusion (V/Q) scans were used if there was a contraindication for CT pulmonary studies. Patients were stratified by PE severity using the AHA classification scheme. Patients were classified as having a massive PE if clinical notes or vitals recorded cardiac arrest, systolic blood pressure <90 mmHg for >15 min, or vasopressor requirement prior to intervention. For the purposes of classifying sub-massive PE, right heart strain was assessed as reported in the CT or echocardiogram at time of presentation. Evidence of cardiac injury was assessed by elevated serum laboratory values of troponin or brain natriuretic peptide.

### CDT procedure description

CDT was performed with either the Unifuse (Angiodynamics) or Cragg-McNamara (Medtronic) conventional infusion catheters. After venous access was obtained using ultrasound guidance, initial pulmonary artery manometry and angiography were performed. Infusion catheters were then placed in the pulmonary arteries under direct fluoroscopic guidance. Patients with single catheter placement received recombinant plasminogen activator (rtPA) (Activase; Genentech) at 0.5–1 mg/h, while patients with two catheters received 1.0 mg/h divided between catheters. Systemic anticoagulation with heparin was achieved using a low-dose thrombostabilizer protocol with a target activated partial thromboplastin time (aPTT) of 53–68 s. Follow-up pulmonary artery manometry and angiography were performed at 12- or 24-h intervals after initiation of CDT at the discretion of the interventional radiologist.

### FTMT procedure description

Anticoagulation was initiated in patients who received FTMT prior to arrival at the procedure room. Additional heparin was given to achieve activated clotting time of 250–300 s. Vasculature was typically accessed via the right femoral vein. A balloon-tipped or pigtail catheter was navigated through the heart into the pulmonary arteries, and pressure measurements were taken. Once the pulmonary vasculature was accessed, the catheter was exchanged for a Triever aspiration catheter (16 Fr, 20 Fr, or 24 Fr), and aspiration was repeated as needed. Mechanical disruption of embolic debris with the FlowTriever disks is not used at our institution. Once it became available (August 2021), the FlowSaver blood return system (Inari Medical) was implemented in the majority of FTMT procedures to filter aspirated blood in the syringe and return it to the patient through the access site. The decision of when to terminate the FTMT procedure was at the discretion of the interventional radiologist based on thrombus removal and observed improvement in clinical status.

### Outcomes & analysis

Primary outcomes included mortality at both 7 and 30 days, procedure-related decompensation (including arrhythmia, cardiac arrest, and major bleeding), and non-procedure-related decompensation (including pneumonia, secondary PE, cancer, stroke, and hemorrhage). Decompensation was considered procedure-related when the events took place from the start of the procedure until the catheter was removed, including arrhythmia, cardiac arrest, and major bleeding as defined by the Society of Interventional Radiology adverse event criteria. The SIR criteria for major bleeding include intracranial, intraocular, or retroperitoneal hemorrhage or any hemorrhage requiring transfusion and/or resulting in a hematocrit decrease ≥15% or hemoglobin decrease ≥5 g/dL. Decompensation events that occurred after the catheters were removed were coded as non-procedure-related. All decompensation events were analyzed by two independent reviewers, with any discrepancies resolved by a third reviewer. These outcomes were compared using the risk ratio (RR) with Fisher's Exact Test for statistical significance.

Secondary outcomes included total length of stay (LOS) post-procedure; ICU LOS; and changes before and after treatment of mean pulmonary artery pressure (mPAP), systemic mean arterial pressure (MAP), and hemoglobin (Hgb). LOS values were measured as the number of overnight stays after the initial PE intervention until discharge from the ICU or from the hospital. A Paired *T*-test was used to evaluate for statistically significant differences in these groups. A two-tailed analysis was used as it is more conservative in identifying differences.

### Ethics statement

This research activity was granted exemption from full Institutional Review Board review and waived informed consent by a qualified staff member of the Human Research Protection Program of the affiliated university in accordance with 45 CFR 164.512(i)(2)(ii).

## Results

### Patient characteristics

In total, 461 patients were included in this retrospective analysis including 308 receiving FTMT and 153 treated with CDT The demographic information for all included patients is summarized in Table 1. Notably, the FTMT population was older on average compared to the CDT group (62.0 ± 16.7 vs. 57.4 ± 16.1; *P* = 0.004). The FTMT group also had higher rates of obesity (63.3% vs. 73.9%; *P* = 0.027) and malignancy (18.8% vs. 9.1%; *P* = 0.006). Whereas the CDT group had more patients with a previous history of PE (11.4% vs. 18.9%; *P* = 0.032).

**Table 1:** Measured demographics of patient presenting with acute PE included in this analysis.

Patient Characteristics	FTMT (*n* = 308)	CDT (*n* = 153)	*P*-value
Age, years	62.0 ± 16.7	57.4 ± 16.1	**0** **.** **004**
Female	145 (47.1)	74 (48.4)	0.843
Body mass index, kg/m^2^	34.9 ± 10.5	35.8 ± 9.0	0.335
Tobacco	72 (23.4)	29 (18.9)	0.339
Type 2 Diabetes	76 (24.7)	33 (21.6)	0.487
Hypertension	184 (59.7)	80 (52.3)	0.135
Obesity	195 (63.3)	113 (73.9)	**0**.**027**
Malignancy	58 (18.8)	14 (9.1)	**0**.**006**
Immobility (>30 d)	83 (26.9)	32 (20.9)	0.171
Recent Surgery (<3 mo)	49 (15.9)	30 (19.6)	0.359
Pregnancy	3 (1.0)	4 (2.6)	0.228
Oral contraceptives/Estrogen	14 (4.5)	10 (6.5)	0.379
Prior deep vein thrombosis	36 (11.7)	28 (18.3)	0.063
Prior Pulmonary Embolism	35 (11.4)	29 (18.9)	**0**.**032**
Massive Pulmonary embolism	23 (7.5)	11 (7.2)	1
Submassive Pulmonary embolism	285 (92.5)	142 (92.8)	1

Data is presented as mean ± SD or number (percent).

Bold values represent *p* < 0.05.

### Procedural characteristics

FTMT was performed by 17 different interventional radiologists who ranged from 1 to 140 thrombectomies each with 90.6% performed by operators with at least 10 thrombectomies. CDT was done by 15 interventionalists with a range of 1–46 procedures each. FTMT was significantly shorter in duration compared to CDT (89.5 ± 31.8 vs. 111.2 ± 44.0 min, *p* < 0.001). Total fluoroscopy time was similar between FTMT and CDT (23.3 ± 10.5 vs. 23.8 ± 13.3 min, *p* = 0.69). The CDT group underwent catheter placement and infusion of local thrombolytics with mean dose of 25.3 ± 11.1 mg and duration of indwelling catheter of 25.8 ± 9.8 h.

### Primary outcomes

There was no significant difference in 7-day mortality comparing FTMT to CDT (RR 0.495, 95% CI: 0.175–1.402, *p* = 0.25). In the FTMT group, 7 (2.3%) patients died in this post-procedure window. Of these cases, 3 (1.0%) had unsuccessful thrombectomy and then coded in the procedure suite or after transfer to the ICU. Of those with successful procedures, 3 (1.0%) later experienced cardiac arrest or neurological damage that led to their deaths. There was 1 (0.3%) case where, after successful thrombectomy, the patient had an apparent hemorrhage and died before a source could be located. The cases of short-term mortality for the CDT group included 3 (2.0%) successful CDT cases who died later in the ICU, 1 (0.7%) case that did not have improvement with CDT, 2 (1.3%) patients that coded on placement or removal of catheters, and 1 (0.7%) patient that died before treatment response could be measured. The majority of cases with 7-day mortality in both FTMT and CDT groups were cases that presented with massive PE.

There was also no significant difference when evaluating 30-day mortality in the FTMT compared to the CDT group (RR 1.347; 95% CI: 0.577–3.12, *p* = 0.67). All deaths in the CDT group occurred in the short-term 7-day mortality window. Deaths in the FTMT group that occurred after 7 days but before 30 days included 9 cases with successful FTMT and 3 cases with partial technical success. Notably, 10 of the 12 cases had confirmed or suspected cancer. The two remaining cases included death from septic shock secondary to COVID pneumonia and a paradoxical stroke through a patent foramen ovale, which led to devastating neurological consequences and withdrawal of life support.

Fewer procedural-related decompensations, including arrhythmias, cardiac arrest, stroke, and major bleeding, occurred in the FTMT group (RR 0.221, 95% CI: 0.069–0.706, *p* = 0.013). Decompensation events that occurred after the catheters were removed were coded as non-procedure-related and are detailed in Table 2. There was no significant difference between the two groups (RR 1.091, 95% CI: 0.530–2.246, *p* = 0.51). Importantly, there was no statistically significant difference in major bleeding events between the two groups. For procedure-related major bleeding, there were no cases in the FTMT group and only 2 in the CDT group that presented as hemoptysis or acute-on-chronic anemia. There was also 1 ICH and 1 non-procedure-related hemorrhage in the FTMT group, while there were none in the CDT group.

**Table 2:** Summary of both procedure-related and non-procedure-related decompensation in both the FTMT and CDT groups.

Outcomes	FTMT (*n* = 308)	CDT (*n* = 153)	RR (95% CI)	*P*-value
7-day mortality	7 (2.3)	7 (4.6)	0.50 (0.18 −1.4)	0.25
30-day mortality	19 (6.2)	7 (4.6)	1.35 (0.58–3.1)	0.67
Decompensation Events	26 (8.4)	19 (12.4)	0.68 (0.39–1.19)	0.19
Procedure-related decompensation	4 (1.3)	9 (5.9)	0.22 (0.069–0.71)	**0** **.** **012**
Arrhythmia	0 (0.0)	1 (0.7)		
Cardiac arrest	3 (1.0)	6 (3.9)		
Major Bleeding	0 (0.0)	2 (1.3)		
Stroke	1 (0.3) (ischemic)	0 (0)		
Non-procedure-related decompensation	22 (7.1)	10 (6.5)	1.09 (0.53–2.25)	0.51
Pneumonia	5 (1.6)	0 (0.0)		
PE	5 (1.6)	7 (4.6)		
Cancer	4 (1.3)	2 (1.3)		
Ischemic Stroke	1 (0.3)	0 (0.0)		
Hemorrhage	1 (0.3)	0 (0.0)		
Other^a^	6 (1.9)	1 (0.7)		
Procedure-related transfusions	*n* = 0 mean pRBCs: 0 ± 0	*n* = 2 mean pRBCs: 2 ± 0		
Total In-hospital Transfusions	*n* = 37 mean pRBCs: 3.4 ± 3.6	*n* = 14 mean pRBCs: 2.3 ± 1.4		

Data is presented as mean ± SD or number (percent).

Bold values represent *p* < 0.05.

a Includes hypotension of unknown etiology, sepsis, congestive heart failure, chemical pneumonitis, and unrelated cardiac arrest.

### Secondary outcomes

**Figure 1:**
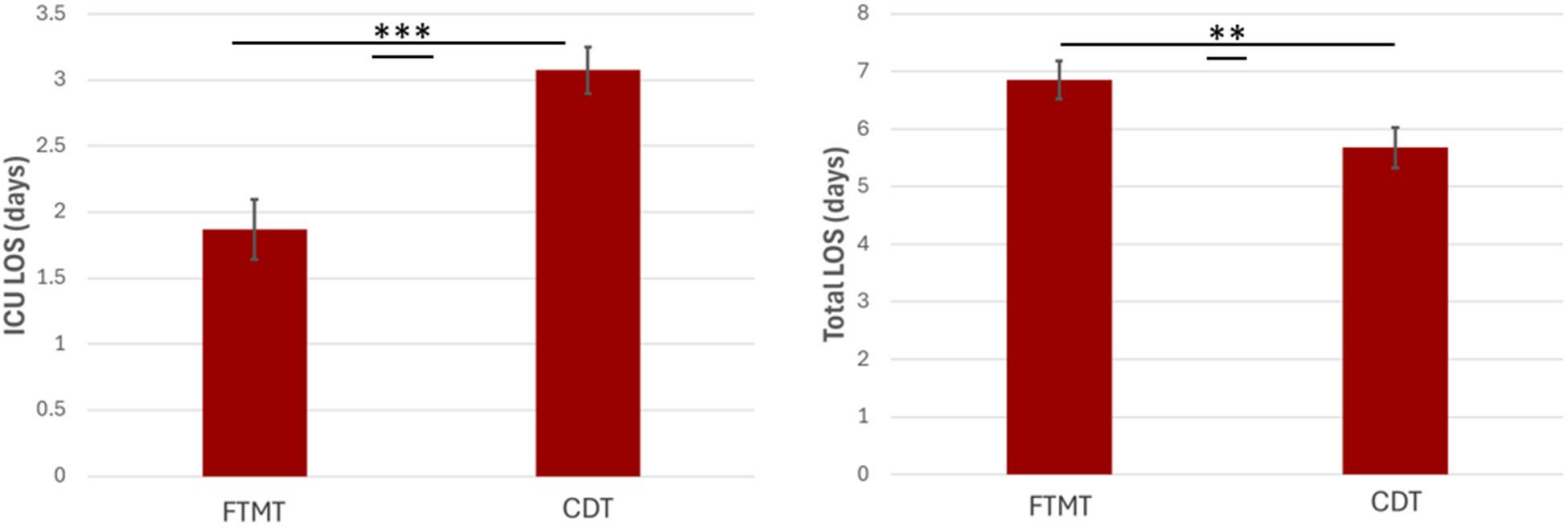
ICU and total length of stays (LOS). **(A)** LOS in ICU for patients after their initial intervention for PE. **(B)** Total LOS following initial FTMT or CDT procedure. Days were counted as overnight stays in each respective department. Bar height represents the mean LOS. Error bars represent the standard error of the mean. ***p < 0.001. **p < 0.01.

Hospital utilization of each group is detailed in Figure 1. There was significantly less ICU utilization in the FTMT group compared with CDT, including both rates of ICU admission (47.7% vs. 97.4%; *P* < 0.001) and ICU LOS (1.87 ± 3.99 vs. 3.07 ± 2.18 days; *p* < 0.001). Despite less ICU utilization, overall LOS was significantly longer in the FTMT group (6.85 vs. 5.68 days; *p* = 0.016).

**Figure 2:**
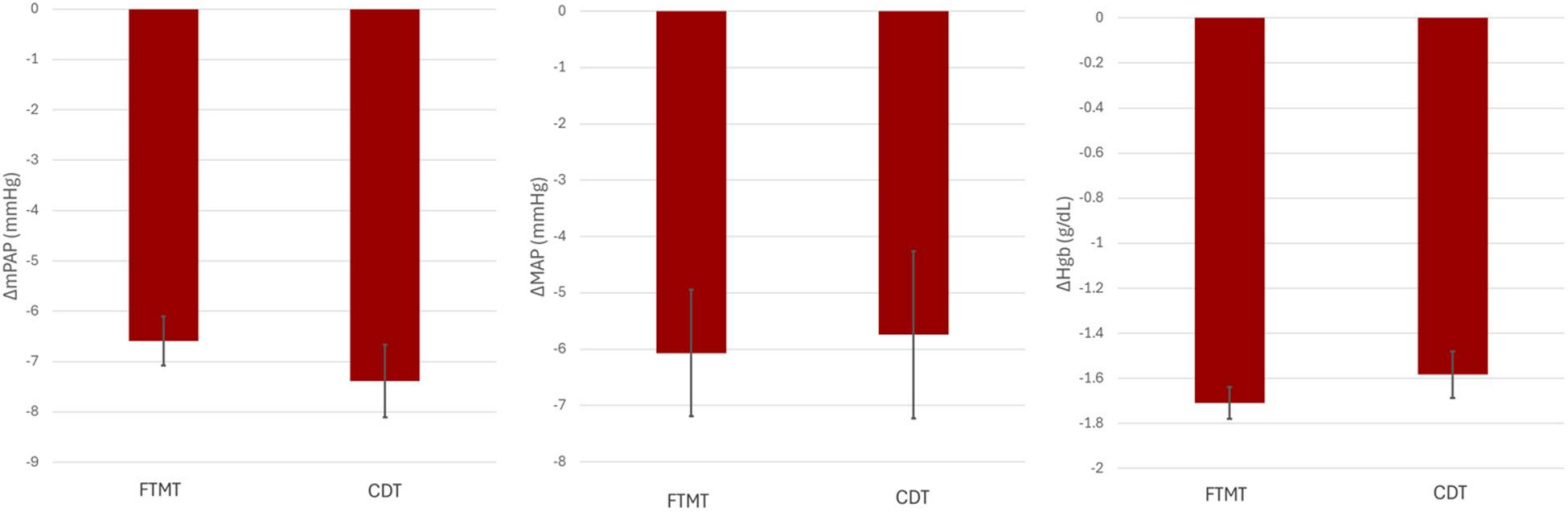
Hemodynamic changes before and after intervention. **(A)** Change in mean pulmonary artery pressure (mPAP). **(B)** Change in mean arterial pressure (MAP). **(C)** Change in Hgb levels. All values were initially measured at the index procedure and again at the conclusion of the treatment.

Additional secondary outcomes for this study include change in systemic MAP, mPAP, and Hgb levels before and after each intervention, as detailed in Figure 2. In both FTMT and CDT, there was a decrease in MAP, but there was no significant difference when comparing the two interventions (−6.07 vs. −5.74; *p* = 0.86). Similarly, there was a decrease in mPAP after each treatment; however, there was no significant difference when comparing the two treatments (FTMT: −6.59 vs. CDT: −7.39; *p* = 0.36). Patients in each group experienced a similar mild decrease in Hgb following treatment (FTMT: −1.71 vs. CDT: −0.95 g/dL; *p* = 0.23).

### Stratified analysis of submassive and massive PE

When the results were stratified between submassive PE (*n*=) and massive PE (). No significant differences were observed between patients presenting with submassive PE treated with either approach. FTMT yielded greater drop in mPAP than CDT in patients presenting with massive PE (−7.43 vs. −0.67; *p* = 0.007). Otherwise no significant differences were observed between the two treatment arms in patients presenting with massive PE.

## Discussion

Reproducing findings of carefully controlled clinical trials with review of outcomes in actual clinical practice is important. Systematic reviews of randomized controlled clinical trials in endovascular intervention have demonstrated inadequate description, standardization, and monitoring of devices, limiting generalizability of findings from randomized controlled trials (17, 18). The literature is particularly limited by a preponderance of small, uncontrolled case series, poor reporting standards, and inadequate monitoring—all of which complicate reproducibility of device performance in actual clinical practice (19–23). The primary outcomes examined in this review of a large cohort of patients treated in routine clinical practice at a single center are mostly in accord with findings of the multicenter, randomized control trial PEERLESS; specifically no significant difference in mortality or major bleeding events between the two therapies, lower occurrence of clinical deterioration/bailout in FTMT group, and less ICU utilization in the FTMT group.

The overall mortality of patients treated with either approach at our institution was significantly higher than that of the PEERLESS study likely due to several factors, the most obvious being that patients presenting with hemodynamic instability were included in this review but excluded from randomization in PEERLESS. Most deaths occurred in patients presenting with massive PE. All patients with a life expectancy of less than 30 days were excluded from PEERLESS. This criterion was not strictly applied at our institution. Finally, patients with any contraindication to thrombolytics were excluded from PEERLESS; however, patients with these factors were routinely treated with FTMT at our institution. Despite these differences in patient characteristics between the two study populations, neither approach outperformed the other in terms of overall mortality.

Similar to PEERLESS, there was no significant difference in the occurrence of major bleeding between FTMT and CDT in our clinical practice. The decrease in serum hemoglobin following each procedure was also not significantly different between treatment approaches. The lower overall event rate of major hemorrhage could be due in part to an information bias of data gathered retrospectively as compared to strict monitoring during a randomized controlled trial protocol. The lack of benefit in terms of major bleeding with FTMT as compared to CDT reported here, in PEERLESS, and other observational series undermines a major rationale behind the development and widespread adoption of mechanical thrombectomy (14–16). A possible explanation is that the bleeding risk present with CDT is not attributable to the use of thrombolytics but simply inherent to the pulmonary artery intervention.

Unlike the PEERLESS trial, there was not a standardized follow up period for patients undergoing PE treatment in this retrospective review. To better relate the occurrence of decompensation events to either therapy, these events were coded based on whether each occurred during therapy (procedure-related) or thereafter (non-procedure-related). There were fewer procedure-related decompensations within the FTMT group. A similar conclusion was reached in PEERLESS, which noted few decompensation events and treatment bailouts in the FTMT group. The lower occurrence of procedure-related decompensation events suggests FTMT to be a safer or more reliable method of removing clot, perhaps because of its immediate effect. However, the difference observed here may reflect an observation bias, as there was a shorter period of time for a decompensation event to occur in the FTMT group with a mean procedure time of 90 min as compared to the mean duration of indwelling catheter of 26 h in the CDT group. Further, most CDT patients were admitted into the ICU and thus under more intense monitoring as compared to those admitted to stepdown ICU or general wards in the FTMT group.

In our institution, thrombolytics are administered within the ICU, whereas the decision of where to admit patients undergoing FTMT is at the discretion of the admitting service. This policy likely explains the lower ICU utilization in the FTMT group more so than a true difference in the level of patient acuity following either procedure. Even still, lower ICU utilization without increased mortality is a benefit of using FTMT as compared to CDT. This finding was also demonstrated in PEERLESS. Interestingly, the total length of stay within the FTMT group was longer. This finding is at odds with PEERLESSS and several other studies (16, 24, 25). The reason for this difference is not entirely clear, but may reflect the greater proportion of patients admitted with cancer in the FTMT group, likely requiring additional imaging, consultation, and ancillary services as part of their initial cancer diagnosis.

This retrospective analysis has several limitations. There was a lack of randomization and, therefore, different baseline characteristics between the treatment groups including differences in average age and in prevalence of obesity, malignancy, and prior PE episodes. Patients with increased bleeding risk could have been preferentially treated with FTMT over CDT, however both treatments were available contemporaneously at our institution for a short period of time. Finally, technical experience gained by interventionalists during the CDT era could have improved their performance during the subsequent era of FTMT-based intervention. This learning may in-part explain the decreased procedure-related decompensations observed here.

## Conclusion

This retrospective review of a large cohort of patients treated with either FTMT or CDT in routine clinical practice supports several findings of the PEERLESS randomized controlled trial, specifically that patients undergoing FTMT utilize less ICU resources and experience fewer procedure-related decompensation events. Overall mortality and major bleeding events between the two therapies are similar.

## Data Availability

The data analyzed in this study is subject to the following licenses/restrictions: dataset contain personal health information and are stored under controlled access at Indiana University. Requests to access these datasets should be directed to pebror@iu.edu.
